# Beet Necrotic Yellow Vein Virus Noncoding RNA Production Depends on a 5′→3′ Xrn Exoribonuclease Activity

**DOI:** 10.3390/v10030137

**Published:** 2018-03-19

**Authors:** Alyssa Flobinus, Nicolas Chevigny, Phillida A. Charley, Tanja Seissler, Elodie Klein, Claudine Bleykasten-Grosshans, Claudio Ratti, Salah Bouzoubaa, Jeffrey Wilusz, David Gilmer

**Affiliations:** 1 Institut de biologie moléculaire des plantes, CNRS UPR2357, Université de Strasbourg, 67084 Strasbourg, France; alyssa.flobinus@gmail.com (A.F.); nicolas.chevigny@etu.unistra.fr (N.C.); tanja.seissler@gmail.com (T.S.); elodie.klein@ibmp-cnrs.unistra.fr (E.K.); salah.bouzoubaa@ibmp-cnrs.unistra.fr (S.B.); 2 Department of Microbiology, Immunology & Pathology, Colorado State University, Fort Collins, CO 80523-168, USA; Phillida.Charley@colostate.edu; 3 SESVanderHave, B3300 Tienen, Belgium; 4 Génétique moléculaire génomique microbiologie, CNRS UMR7156, 67000 Strasbourg, France; bleykasten@unistra.fr; 5 DipSA-Plant Pathology, University of Bologna, 40127 Bologna, Italy; claudio.ratti@unibo.it

**Keywords:** Viral noncoding RNA, exoribonuclease, flavivirus, BNYVV, VIGS

## Abstract

The RNA3 species of the beet necrotic yellow vein virus (BNYVV), a multipartite positive-stranded RNA phytovirus, contains the ‘core’ nucleotide sequence required for its systemic movement in *Beta macrocarpa*. Within this ‘core’ sequence resides a conserved “coremin” motif of 20 nucleotides that is absolutely essential for long-distance movement. RNA3 undergoes processing steps to yield a noncoding RNA3 (ncRNA3) possessing “coremin” at its 5′ end, a mandatory element for ncRNA3 accumulation. Expression of wild-type (wt) or mutated RNA3 in *Saccharomyces cerevisiae* allows for the accumulation of ncRNA3 species. Screening of *S.* *cerevisiae* ribonuclease mutants identified the 5′-to-3′ exoribonuclease Xrn1 as a key enzyme in RNA3 processing that was recapitulated both in vitro and in insect cell extracts. Xrn1 stalled on ncRNA3-containing RNA substrates in these decay assays in a similar fashion as the flavivirus Xrn1-resistant structure (sfRNA). Substitution of the BNYVV-RNA3 ‘core’ sequence by the sfRNA sequence led to the accumulation of an ncRNA species in yeast in vitro but not in planta and no viral long distance occurred. Interestingly, XRN4 knockdown reduced BNYVV RNA accumulation suggesting a dual role for the ribonuclease in the viral cycle.

## 1. Introduction

In eukaryotic cells, the RNA decay machinery plays a major role in determining both the quantity and quality of gene expression. Nuclear RNA surveillance complexes remove aberrant and unprocessed transcripts, while the cytoplasmic RNA decay machinery coordinates with translation to determine the half-life of mRNAs [1]. Most mRNA degradation is initiated by deadenylation followed by either decapping and 5′-3′ degradation by the highly processive Xrn family of exoribonucleases or in a 3′-5′ pathway via exosome-mediated degradation [2]. Viral transcripts are also significantly impacted by these RNA decay pathways. Therefore, viruses have evolved different strategies to control and stabilize their messengers or genome components. These include cleavage of decay-promoting factors, such as AUF1, by picornaviruses [3], recruitment of the mRNA stabilizing protein HuR by alphaviruses [4], or repression of the decay machinery itself (for reviews see [5,6,7]).

The process of subverting the host RNA decay machinery can also lead to the generation of new viral RNA species. Arthropod-borne flaviviruses use a knot-like three helix junction structure at the beginning of the 3′ untranslated region of their genomic RNAs to stall Xrn1 and generate a stable RNA decay intermediate [8,9,10,11,12,13]. This sfRNA enhances viral pathogenicity [9,14,15] and inhibits the RNA silencing machinery [16,17]. In plants, red clover necrotic mosaic virus also uses Xrn stalling to produce SR1f, an ncRNA species that influences the translation of both viral and cellular RNAs. Despite the highly processive nature of Xrn enzymes that can easily degrade through structured transcripts such as rRNA, relatively small (~50–75 nts) but apparently highly specialized structures in viral transcripts can stall the enzyme [18] In SR1f, for example, a 58 nt sequence is required to protect the RNA from 5′-to-3′ degradation [19].

Animal, insect, and yeast Xrn1 homologs are cytoplasmic RNA decay enzymes that ensure proper homeostasis and cell growth [20]. These enzymes have a 5′-to-3′ exoribonuclease activity and target 5′-monophosphorylated RNAs that are released after endoribonuclease cleavage or by the action of Dcp2 or related decapping enzymes [21,22]. In plant cells, whereas mRNA decay involves Dcp2 decapping, 5′-to-3′ processing requires the exoribonuclease XRN4, a yeast Xrn2 ortholog with a cytoplasmic localization and Xrn1-like function [23]. XRN4 activity has been implicated in plant defense against viruses because its overexpression is associated with an increase in viral RNA degradation in *Nicotiana benthamiana* [24]. Furthermore, silencing of *NbXRN4* enhances viral accumulation in local infections of tobacco mosaic virus (TMV) and accelerates its systemic movement in the plant [25], while an opposite effect is described for bamboo mosaic virus (BaMV) for which XRN4 is associated with the replication machinery [26].

Beet necrotic yellow vein virus (BNYVV) belongs to the *Benyviridae* family and is the type member of the *Benyvirus* genus [27,28]. BNYVV is transmitted by the soil-borne protozoa *Polymyxa betae* and causes the sugar beet Rhizomania disease. The BNYVV genome is composed of four to five linear, positive-sense, single-stranded, capped, and polyadenylated RNAs. The small viral genomic RNAs are required for the natural infection of *β* species and for transmission of the virus, but are dispensable for laboratory host mechanical infection since BNYVV-RNA1 and -RNA2 are necessary and sufficient for viral replication in rub-inoculated laboratory host plants [29]. RNA1 encodes the protein required for replication, while RNA2 ensures the expression of proteins needed for packaging, cell-to-cell movement, and RNA silencing suppression. Besides its function as a viral suppressor of RNA silencing (VSR), the p14 protein is also required for the systemic spread of the virus [30], acting synergistically with the subgenomic ncRNA3 accumulation for viral long-distance movement [31]. The ncRNA3, initially described as RNA3sub [32], is processed by a cellular enzymatic activity [33] and is essential for viral long-distance movement in *β* species*.* Systemic infections of *β macrocarpa* absolutely require a 20 nt long “coremin” sequence that is embedded in the RNA3 ‘core’ region [33,34,35]. In the absence of evidence for a subgenomic promoter, ncRNA3 is thought to be generated by post-transcriptional processing. However, the enzyme responsible for ncRNA3 production has not been identified to date.

Here, we use genetic approaches in *Saccharomyces cerevisiae* to show that ncRNA3 production can be achieved by the action of either the yeast exoribonuclease Xrn1 or plant XRN4. The coremin motif embedded within a 55 nt region is sufficient to stall 5′-to-3′ exoribonucleases in vitro and in insect cell extracts similar to the sfRNA structural motif. The ‘core’ domain substitution with the sfRNA sequence allows the accumulation of ncRNA species in yeast and in vitro. However, such substitution surprisingly prevents the production of ncRNA in the viral context as well as long-distance movement of the chimeric virus in plants. These data suggest that the original “coremin” sequence itself, rather than simply stalling Xrn-mediated RNA decay, is mandatory for viral systemic movement in *Beta macrocarpa.* Virus-induced gene silencing (VIGS) of *NbXRN4* downregulated BNYVV-RNA accumulation, a situation that recalls bamboo mosaic virus behavior [26] without obvious effects on the ncRNA production.

## 2. Materials and Methods

### 2.1. Plasmids

The *Eco*RI restriction site (position 377) of the full-length cDNA clone of BNYVV-RNA3 (pB35) was filled in with a Klenow fragment then self-ligated to produce pB36. pB36 served for site-directed mutagenesis [36] to insert an *Eco*RI site at the 5′ terminus of the genome to produce a pB37 infectious clone The resulting cDNA copy of RNA3 was then cloned into *Eco*RI-*Hin*dIII-digested p426GPD vector to produce p426GPD37. The fragment from pB35E containing the mutated “coremin” sequence [33] was subcloned into p426GPD37 using *Bam*HI-*Hin*dIII restriction sites to create the p426GPD37E vector.

A BN3sf cDNA clone and its BN3pk1 variant were obtained as synthetic genes products ordered at Genscript® (Piscataway, NJ, USA) and delivered into pUC57 between *Eco*RI and *Hin*dIII restriction sites. BN3 and 1R to 9R constructs contained an *Eco*RI and a T7 promoter sequence followed by the BNYVV-RNA3 3′ end cDNA sequence starting from *Bam*HI (position 750) to *Hin*dIII (BN3, located after the poly A tail or 1R to 9R, between positions 1739 to 1308). BN3sf/pk1 differed from BN3 by the nt 1147–1477 replacement with nt 10,422–10,728 from the flaviviral sequence (accession number AY274505). The vectors BN3sf and BN3pk1 served for a *Bam*HI-*Hin*dIII sequence switch with p426GPD37 and pB35 to produce p426GPD-RNA3sf/pk1 and pB35sf/pk1, respectively.

A plasmid encoding the BNYVV-55 element (sequences 1222–1277 from BNYVV RNA3) was generated by inserting oligos containing the indicated BNYVV sequence (NBCI reference sequence: NC_003516) into the *Pst*I and *Hin*dIII of pGEM-4 (Promega, Madison, WI, USA). The plasmid used to produce the RNA containing the upstream portion of the DENV 3′ UTR has been described previously [37]. Internally radiolabeled, 5′ monophosphorylated RNAs were generated using SP6 polymerase as described previously [37] and purified on denaturing acrylamide gels prior to use. To generate the BNYVV-55mer (also called B-3 in Figure 6), the parent plasmid was linearized with *Hin*dIII. The plasmid was linearized with *Ear*I to generate competitor RNAs. The BNYVV variant B-1 and B-2 RNAs were generated from plasmids linearized with *Hyp*CH4III and *Hyp*1881, respectively. Control RNAs were generated from pGEM-4 linearized with either *Ear*I (to generate 168 base cold competitor RNA) or with *Hin*dIII (to generate the 61 base radiolabeled ‘reporter’ RNA in Figures 6 and 7). The DENV 3′ UTR plasmid was linearized with *Ear*I to generate the DENV 3′ UTR competitor RNA.

Yeast vectors pRS415 (empty backbone), pAJ152 (expressing Xrn1), and pRDK307 (expressing a catalytic mutant of Xrn1) were provided by Lionel Benard [38]. Yeast vector pAG423GPD-XRN4 expressing *Arabidopsis thaliana* XRN4 (ATXRN4) was obtained by Gateway cloning between pDONR207 containing *A. thaliana XRN4* gene (http://www.addgene.org/vector-database/2393/) and the destination vector pAG423GPD-ccdB HA (https://www.addgene.org/14246/), using LR Clonase™ enzyme mix (Invitrogen, Carlsbad, CA, USA) following the manufacturer’s recommendations.

### 2.2. Yeast Transformation

Yeast strains were cultured on YPD complete media and transformed using the method described by Gietz and Schiestl [39]. Transformed yeasts were selected onto appropriate synthetic media depleted with uracil, or uracil and histidine (pAG423GPD-XRN4). Yeast total RNAs were extracted using TRIzol reagent (Invitrogen) following the manufacturer’s recommendation.

#### In Vitro Transcription, Plant Infection, Protein, and RNA Extractions

In vitro production of ncRNA3 was from a pUC19 construct containing the ncRNA3 sequence (nt 1234 to 1774 followed by 15A) downstream of a T7 promoter. Amplicons allowing the synthesis of transcripts possessing 3′ shortened ends were from T7 promoter-containing primer starting position 750 together with primers complementary to RNA3, ranging from position 1739 to 1308 (Figure 5). The linearized full-length BNYVV clones of RNA1 (pB15), RNA2 (pB21), RNA2Δp14 (pB2-3722), RNA2BA2 (pB2-BA2) [30], RNA3 (pB35), RNA3E (pB35E), and the recombinants RNA3sf (pB35sf) or RNA3pk1 (pB35pk1) served for in vitro run-off transcription as described previously [31].

RNA1 and RNA2 transcripts supplemented or not with wild-type (wt), mutated, or chimeric RNA3 served for the mechanical infection of *Chenopodium quinoa* and *B. macrocarpa* or for the electroporation of *C. quinoa* protoplasts as described before [40,41,42]. RNA extractions were performed from infected *C. quinoa* local lesions and protoplasts. RNAs were extracted using “Polysomes” buffer [43] followed by phenol/chloroform extraction and ethanol precipitation. Pellets were treated with 3 M sodium acetate solution (pH5.5) to solubilize DNA and small RNAs. Remaining RNA pellet was washed with ethanol (70%) before dissolution in sterile water. RNAs were extracted from protoplasts after cell lysis in suspension buffer (Tris-HCl 50 mM, pH7.5, EDTA 1 mM, Macaloïde 0.05%, SDS 1%, NaCl 150 mM) followed by two extractions with phenol/chloroform and ethanol precipitation. Northern blot and 5′RACE experiments were performed as described previously [33].

### 2.3. Virus-Induced Gene Silencing in Nicotiana benthamiana or Gene Silencing Experiments in Nicotiana benthamiana

Cultures of *Agrobacterium tumefaciens* cells (GV3101 or C58C1) were prepared as described previously [44] for agroinfiltration of *N. benthamiana* (4–5 weeks-old seedling). XRN4 gene silencing was performed as described [26] and VIGS was monitored visually in parallel on plants infected with tobacco rattle virus (TRV) carrying a Phytoene desaturase (PDS) fragment. Eleven days post infiltration, when the effect of the PDS gene silencing was visible, silenced apical leaves were infected with BNYVV-RNA1 and RNA2BA2 mutant [31] supplemented with RNA3 or RNA3E. After 21 days, RNAs were extracted from inoculated and systemic leaves using TRI reagent® (Molecular Research Center, Cincinnati, OH, USA) following the manufacturer’s recommendations.

### 2.4. Reverse Transcription qPCR

RNAs were treated with RQ1 DNAse (1 U, Promega) before reverse transcription using oligo(dT) primer (Thermo Scientific, Waltham, MA, USA) and Improm-II^TM^ Reverse Transcriptase (Promega) following the manufacturer’s instructions. cDNAs were submitted to qPCR using LightCycler^®^ 480 SYBR Green Master mix (Roche, Basel, Switzerland) and a LightCycler^®^ 480 instrument (Roche). Xrn4 5′ and 3′ sequences flanking the VIGS-targeted sequence were assayed using Glyceraldehyde-3-phosphate dehydrogenase (GAPDH) and Protein phosphatase 2A (PP2A) as internal references.

### 2.5. In Vitro Xrn1 Assays

*Hin*dIII-linearized pB35sf, pB35pk1, BN3, BN3sf, and BN3pk1 or *Psi*I-linearized pB35 and pB35E plasmids served to produce in vitro run-off transcripts without adding a Cap analog [29]. The DNA templates were eliminated by a DNase treatment followed by phenol/chloroform extraction and ethanol precipitation. For some experiments, RNAs were precipitated with lithium chloride (2M). The 5′ pyrophosphate was removed by a pyrophosphatase hydrolase treatment (RppH, New England BioLabs, Evry, France) for 1.5 h at 37 °C according to the manufacturer’s recommendations followed by phenol/chloroform extraction and ethanol purification. Recombinant RNA3 transcripts (40 ng), bearing 5′ monophosphate, were incubated with 1 U of commercial Xrn1 (New England BioLabs, Evry, France) for 6 to 12 h at 37 °C. At the time indicated, aliquots of the reactions were stopped in 8 M urea solution (1:1) and RNAs were analyzed by northern blot. When indicated, purified transcripts (3 µg) were incubated simultaneously with commercial RppH (2 U) and Xrn1 (4 U) for 6 h at 37 °C and 2.1 µg of RNA3E were added in the RNA3- and RNA3E-containing reaction tubes. Kinetics was followed for 12 h. Parallel experiments were carried out with a mixture of wt RNA3 (3 µg) and RNA3E (3 µg) in the presence of 4 U of RppH and 8 U of Xrn1.

### 2.6. Cell-Free Xrn1 RNA Decay Assays

In vitro 5′-to-3′ Xrn1 decay assays and competitions were performed essentially as described [12] using C6/36 cytoplasmic extracts [45] under conditions that favor 5′-to-3′ decay [46] or with recombinant *Kluyveromyces lactis* Xrn1 (residues 1–1245) [47] purified from in *Escherichia coli* BL21 cells. For Xrn1 decay assays performed to identify decay intermediates, approximately 85 fmol of each radiolabeled input RNA was used. For RNA competition assays, ~85 fmol of a pGEM-4 reporter RNA was incubated with a 20-fold molar excess of the pGEM-4 control competitor or the viral-derived DENV 3′UTR or BNYVV 55mer core RNAs. These competitor RNAs were lightly radiolabeled with ^32^P-UTP to allow for easy quantification and follow their fate following incubation. RNA reaction products were separated on 5% denaturing polyacrylamide gels and visualized by phosphorimaging. The average of the percent RNA remaining in each time point from three independent experiments was used to generate graphical data. Significance was determined using a two-way ANOVA and *p*-values were generated using Tukey’s multiple comparisons test as a post-hoc test. The * represents a *p*-value <0.001 at both time points for the viral 3′ UTR compared to the control.

## 3. Results

### 3.1. Expression of BNYVV-RNA3 in Yeast Leads to ncRNA3 Accumulation

**Figure 1:**
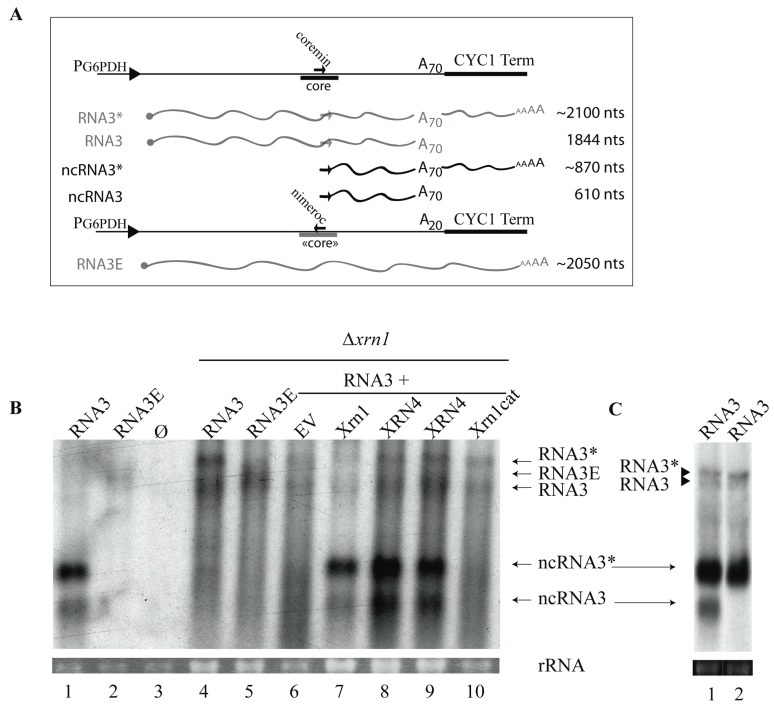
Exoribonucleases are responsible for noncoding RNA3 species accumulation in *Saccharomyces cerevisiae*. (**A**) Representation of the expression vector cassettes (solid lines) used to produce RNA3 and RNA3E species under the control of constitutive G6PDH promoter (black arrowhead). Capped (•) full-length RNAs and ncRNA species are depicted by waved grey and black lines, respectively. Wild-type (wt) ‘core’ sequence is presented in dark and mutated “core” in grey bold lines. The “coremin” motif and its antisense orientation, “nimeroc”, are depicted by black sense and antisense arrows, respectively. Drawings are not to scale and for a better representation of RNA3 species, refer to figure 2 of reference [27]. RNA sizes are presented on the right; (**B**,**C**) Northern blot analyses of RNA3 and ncRNA species produced in yeasts; (**B**) vectors producing wt RNA3, mutated RNA3E, or control vector (Ø) were introduced in *S. cerevisiae* (lanes 1–3) or Xrn1 defective strain (Δ*xrn1*) (lanes 4–5). Expression of RNA3 (lanes 6–10) was performed in ∆*xrn1* strain together with an empty vector (EV, lane 6) or vectors expressing: yeast Xrn1 (lane 7), plant ATXRN4 (XRN4, lanes 8 and 9), or a defective Xrn1 enzyme (Xrn1cat, lane 10). Total RNAs were subjected to northern blot analysis using a beet necrotic yellow vein virus (BNYVV)-specific 3′ probe complementary to nt 1277–1774; (**C**) total RNAs from yeast expressing RNA3 were differentially visualized using two probes (lanes 1 and 2). RNA3, RNA3*, ncRNA3, and ncRNA3* were revealed with a coremin-specific probe (lane 1) while RNA3* and ncRNA3* appeared only when the *CYC1* terminator-specific probe was used (lane 2). Black triangles indicate the two full-length RNA3 species. Loadings were visualized by ethidium-bromide staining (rRNA).

**Figure 2:**
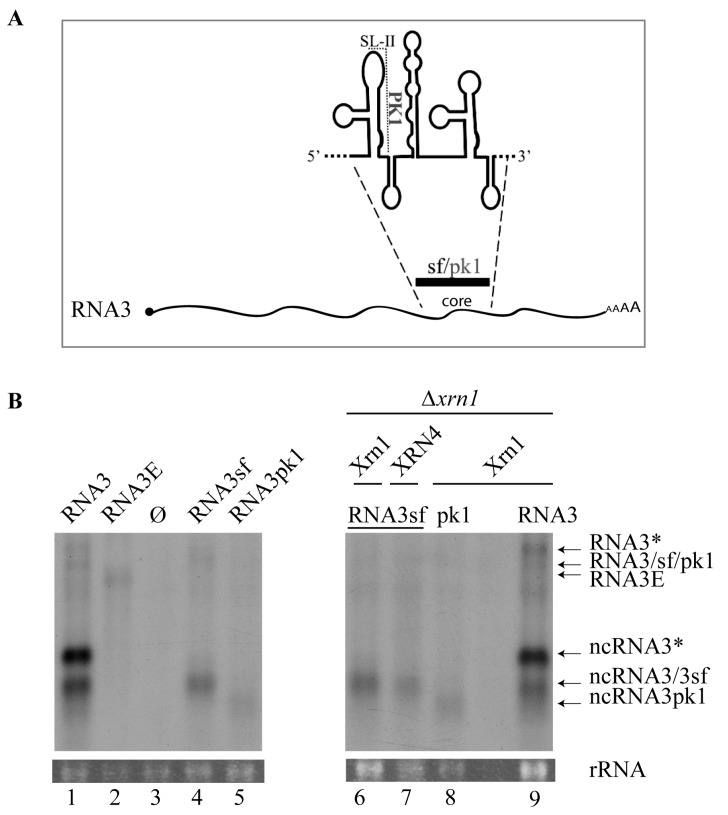
Accumulation of noncoding RNA3sf from chimeric RNA3sf inhibits Xrn1 and AtXRN4 exoribonucleases in *Saccharomyces cerevisiae*. (**A**) The ‘core’ sequence was replaced by a wt (sf) or mutated (pk1) flavivirus sequence to produce RNA3sf and RNA3pk1, respectively. PK1 pseudoknot involving stem-loop II (SL-II) is shown; (**B**) northern blot analyses of RNA3 and ncRNA species produced in yeasts. Plasmids allowing the expression of RNA species indicated that RNA3, RNA3E, RNA3sf, RNA3pk1, or empty vector (Ø) were introduced in wt yeasts (lanes 1–5) or Xrn1-defective yeasts (Δ*xrn1*, lanes 6–9) complemented with a vector allowing for the production of yeast Xrn1 (lanes 6, 8, and 9) or plant XRN4 (lane 7). The RNA3* and ncRNA3* are similar to RNA3 and ncRNA3, respectively, but possess a *CYC1* terminator sequence followed by a polyA tail (see Figure 1 and text for details). Positions of the RNA species are indicated on the right. Total RNAs were subjected to northern blot analysis using a BNYVV-specific 3′ probe complementary to nt 1277–1774. The partial complementarity of the probe with the ncRNA3sf species does not allow for quantitative comparisons. Loading was visualized by ethidium-bromide staining (rRNA). No sample was loaded between lanes 8 and 9.

We previously showed that cellular proteins (nucleases) were implicated in BNYVV-RNA3 processing, leading to ncRNA3 accumulation in a heterologous, non-viral wheat germ extract system [33]. To further explore ncRNA3 biogenesis, *Saccharomyces cerevisiae* FY4 strains were transformed with plasmid constructs expressing either RNA3 deleted of its 5′ UTR (Figure S1A), wt RNA3, or RNA3E, a mutated form of RNA3 (Figure 1A and Figure S1A) in which the “coremin” motif was switched into its antisense orientation, namely “nimeroc”. Due to construction constraints, the poly(A) tail of RNA3E was reduced to 20 nt (Figure 1A). The empty vectors (Ø) were used as controls. Yeast total RNAs were extracted and analyzed by northern blot using a probe complementary to the 3′-conserved RNA3 (Figure 1B and Figure S1B). In the wild-type FY4 strain, the full-length species of RNA3 and RNA3E were hardly visible (Figure 1B and Figure 2B, lanes 1, 2) and ncRNA3 accumulation was only observed in the strain expressing RNA3 (Figure 1B and Figure 2B, lane 1). This experiment suggested that RNA3 species were degraded in yeast and that the RNA3 “coremin” motif was able to prevent its degradation while the “nimeroc” sequence was not, as observed previously in wheat germ extracts [33]. Surprisingly, when RNA3 was expressed, two ncRNA3 species appeared: one species of the expected size (ncRNA3) and another extended species (ncRNA3*) (Figure 1A,B lanes 1, 7–9; Figure S1). We performed 5′ RACE and found that both ncRNA3 species had the same 5′ extremity that is sensu stricto identical to ncRNA3 produced during viral infection [33]. We concluded that full-length RNA3 was expressed as two species differing in their 3′ termini (see below). Northern blots were performed on total RNA extracted from the strain FY4 expressing RNA3 using radiolabeled oligonucleotides either complementary to the “coremin” sequence (Figure 1C, lane 1) or complementary to the *CYC1* terminator sequence (Figure 1C, lane 2). Both ncRNA3 species were detected with the “coremin”-specific probe while only the larger ncRNA (ncRNA3*) was detected with a probe complementary to the *CYC1* terminator sequence, indicating that these two ncRNA3 species indeed differed at their 3′ termini as depicted in Figure 1A. Full-length RNA3 (depicted in Figure 1A) was also observed with these two probes, appearing as two species when detected with the viral antisense probe (black triangles, Figure 1C, lane 1) and as one species when detected with the viral terminator probe (Figure 1C, lane 2), respectively, due to two possible transcription termination sites.

### 3.2. 5′→3′ Exoribonuclease Activity is Responsible for ncRNA3 Accumulation in Yeast

Because the *S. cerevisiae* cellular machinery recapitulates accumulation of ncRNA3 observed in planta, we screened a collection of viable nuclease mutants to identify gene products responsible for RNA3 processing. Four strains were selected, FY4∆*rnh1*, ∆*rrp6*, ∆*rex3*, and ∆*xrn1*, and then transformed with the RNA3 construct. Rnh1 is a nuclease protein implicated in RNA degradation from RNA-DNA duplexes [48]. Rex3 is a 3′-to-5′ exoribonuclease involved in the maturation of the RNase MRP complex [49]. Rrp6 is a 3′-to-5′ exoribonuclease that belongs to the exosome complex implicated in 3′-end maturation of stable RNAs and in the degradation of transiently expressed non-coding RNAs [50]. Xrn1 was described above [21,22]. Total RNA from transformed yeast strains was assayed by northern blotting to detect RNA3 and sizes compared with viral RNAs (Figure S1). Only FY4∆*xrn1* accumulated full-length RNA3 and RNA3E (Figure S1 and Figure 1B, lanes 4 and 5) and traces of ncRNA3 species (Figure 1B, lanes 4, 6). These traces of ncRNA3, also detected by 5′ RACE, probably originated from the nuclear exonuclease activity of Xrn2 (*RAT1* gene product) on nuclear primary transcripts. Unfortunately, the ∆*xrn2* mutant is not available and the double ∆*xrn1*-∆*xrn2* mutant is not viable and could thus not be tested [51]. We then performed complementation assays in ∆*xrn1* yeast strains by co-transforming them with plasmids expressing wt or mutated yeast Xrn1 (Xrn1 or Xrn1cat, respectively [38]), *A. thaliana* XRN4, or an empty vector as control (EV). In the presence of an empty vector, only traces of ncRNA3 species were detected (Figure 1B, lane 6). The ncRNA3 accumulation was restored when functional Xrn1 or XRN4 was provided in trans (Figure 1B, lanes 7, 8, and 9) but not when a non-functional exoribonuclease, Xrn1cat, was used (Figure 1B, lane 10).

Taken together, these results demonstrate that ectopic expression of yeast Xrn1 or plant XRN4 can functionally complement 5′-to-3′ processing of RNA3, leading to ncRNA3 accumulation in vivo. The 5′ extremity of the ncRNA3 species produced by XRN4 complementation in yeast was found to be identical to those characterized in planta, in wild-type FY4, and in Xrn1-complemented yeast strains. Similar experiments were performed using yeast strain W303-1A and mutants ∆*dcp2*, ∆*xrn1*, and ∆*dcp2*-∆*xrn1* [52]. Only wild-type W303-1A was able to produce ncRNA3, while all of the mutants accumulated only RNA3/3E species, indicating the essential role of Dcp2 prior to Xrn1-dependent processing.

### 3.3. Substitution of the BNYVV-RNA3 Core Sequence by the Xrn1-Resistant West Nile Virus Core Sequence Produces a New ncRNA3 in S. cerevisiae Strains Expressing Xrn1 or Arabidopsis thaliana XRN4

Degradation of West Nile virus (WNV, Flavivirus) genomic RNA, including the Kunjin virus strain, provokes an Xrn1 stalling event leading to sfRNA accumulation. Such sfRNA species promote pathogenesis due to their complex secondary folding structure [8,9,12]. The sfRNA 5′ proximal hairpin structure (SL-II) is involved in the formation of the PK1 pseudoknot, which is abolished by three SL-II nucleotide substitutions in the pk1 mutant [11]. Such substitutions prevent the accumulation of sfRNA and lead to the accumulation of distinct ncRNAs corresponding to sfRNA2 [11]. Because the BNYVV-RNA3 ‘core’ sequence and, particularly, the “coremin” motif were shown to be implicated in ncRNA3 stabilization [33], we decided to produce a viral chimeric RNA3 in which the entire ‘core’ sequence was replaced by 307 nt from the Kunjin virus sequence (nt 10,422 to 10,728 from accession number AY274505) or its pk1-mutated version to create RNA3sf and RNA3pk1, respectively (Figure 2A). These two chimeras were used to perform a comparative and translational approach with previously published data. Chimeric RNAs (RNA3sf, RNA3pk1), RNA3, and RNA3E had similar viral sequence sizes and were expressed in the *S. cerevisiae* FY4 strain (Figure 2B, left) and in the FY4∆*xrn1* mutant complemented with *Xrn* genes (Figure 2B, right). It is worth noting that RNA3 possessed a ~70 polyA sequence preceding the terminator sequence while, like RNA3E (Figure 1A), chimeric RNA3sf/pk1 had only 20 A residues due to synthetic construct constraints. As expected, northern blot analyses revealed the accumulation of ncRNA3 when RNA3 was expressed (Figure 2B, lane 1) and not when RNA3E was produced or when an empty vector was used (Figure 2B, lanes 2 and 3). Expression of RNA3sf resulted in the accumulation of a new ncRNA species together with a faint detection of full-length RNA3 species (Figure 2B, lane 4). A 5′ RACE analysis of ncRNA3sf identified the sequence **_5′_GGA**AGUCAGGCCGGAAA…_3′_ that corresponds to the 5′ terminus of sfRNA species extended by three upstream viral residues (bolded) as compared to the published sfRNA 5′ extremity, which has been determined previously by primer extension [9]. Expression of RNA3pk1 resulted in a faint detection of the full-length RNA and the accumulation of a shorter ncRNA species (Figure 2B, lane 5) with a size consistent with the one described by Funk and colleagues [11]. The ncRNA3pk1 5′ extremity has not been characterized in this study. RNA3 and RNA3sf constructs were introduced in FY4∆*xrn1* together with vectors expressing Xrn1 (Figure 2B, lanes 9 and 6, respectively) or XRN4 (Figure 2B, lane 7). FY4∆*xrn1* expressing the RNA3sf construct could not be obtained, probably due to the inhibition of Xrn2. The RNA3pk1 construct was introduced only in FY4∆*xrn1* complemented with the Xrn1-producing vector (Figure 2B, lane 8). The expected ncRNA species were detected in FY4∆*xrn1* complemented either with Xrn1 or XRN4 (Figure 2B, lanes 6 and 7), suggesting that the action of sfRNA on yeast Xrn1 [9] occurs similarly on XRN4 expressed in yeast.

### 3.4. Xrn1 Produces ncRNA3 In Vitro

**Figure 3:**
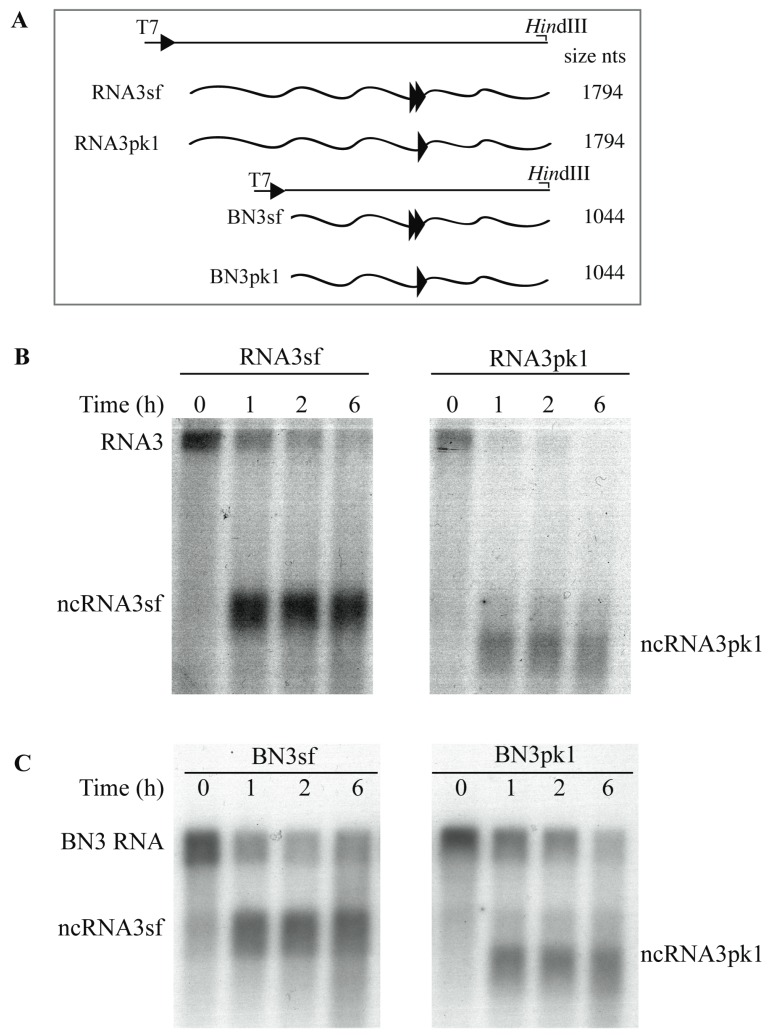
Both ncRNA3sf and ncRNA3pk1 stall Xrn1 processing in vitro. (**A**) Schematic representation of the T7-driven cDNA clones constructs used to produce run-off in vitro transcripts depicted in waved lines. Double and single arrowheads correspond to the sf and pk1 structural motifs, respectively. The position of the restriction sites used and the size of the transcripts are indicated; (**B**) 5′ phosphorylated chimeric RNA3sf or RNA3pk1 and (**C**) BN3sf or BN3pk1 species were mixed with commercial Xrn1 enzyme for 6 h. Aliquots were sampled at the time indicated and RNAs species were detected by northern blot using a specific DNA probe able to reveal both full-length and ncRNA3 species.

We confirmed Xrn1 function in ncRNA production using in vitro transcripts and purified enzymes by adapting a biochemical assay described for flaviviruses [13,18]. Full-length (Figure 3A, RNA3sf and RNA3pk1) or truncated (the first 750 nts 5′ extremity) chimeric transcripts (Figure 3A, BN3sf and BN3pk1) were treated with pyrophosphatase and subjected to Xrn1 degradation. In our hands, the use of a commercial Xrn1 enzyme set the reaction time from 0 to 6 h (Figure 3B,C).

Using chimeric sfRNA substrates (full-length RNA3sf and truncated BN3sf species), we reproduced the accumulation of ncRNA species of expected size (Figure 3B,C, left panels), which was able to block Xrn1 degradation of full-length RNA species as described in [12] 2 h post-treatment. As observed in the yeast experiments above, Xrn1 action on the pk1-mutated sfRNA produced a reduced-sized ncRNA3pk1 (Figure 3B,C, right panels) consistent with previous reports [11,12]. In our hands, the inhibitory effect of the ncRNA3pk1 species appeared to be less efficient compared with wt, as the amounts of full-length RNA and ncRNA3pk1 species decreased over time (Figure 3B,C, compare left and right panels). These results also indicate that the first 750 nt of RNA3 have no effect on ncRNA production.

### 3.5. A Minimal Sequence of 43 nt within the ncRNA Sequence is Sufficient for Xrn1 Stalling

**Figure 4:**
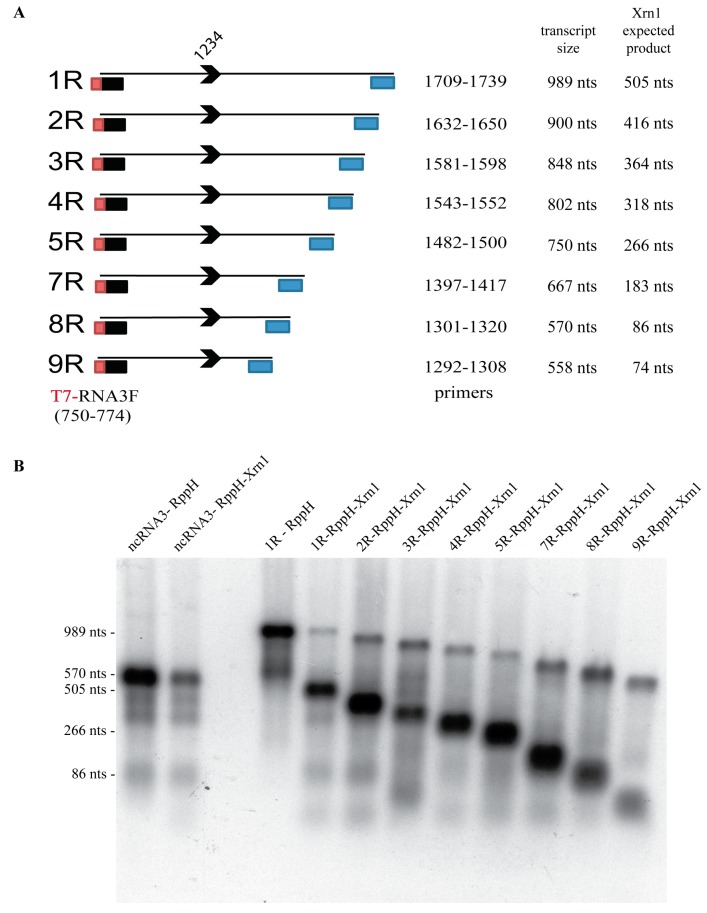
Characterization of the minimal RNA3 3′ domain required for the efficient stalling of the Xrn1 enzyme. (**A**) Representation of the fragments obtained using T7 promoter containing primer (T7-RNA3F) and reverse primers (positions specified by blue boxes) cloned into pUC57 that served as templates for the production of 1R to 9R transcripts, ranging from 989 to 558 nt, possessing the same 5′ extremity with decreasing 3′ end length. The arrowhead locates the 5′ position of the ncRNA3 species (nt 1234). The expected product size after RppH and Xrn1 treatments are specified. Construct 6R was not obtained; (**B**) after 6 h of RppH/Xrn1 treatment, RNAs were analyzed by a 24-cm long run on 1.5% denaturing-agarose gel followed by northern blot using a radiolabeled DNA oligomer probe complementary to the “coremin” sequence. The ncRNA3 transcripts and 1R transcripts were treated with RppH alone or with RppH and Xrn1. The lengths of some RNA species are specified on the left side.

Next, we examined the minimal sequence required for ncRNA accumulation in our in vitro Xrn1 resistance assay. For this purpose, in vitro transcripts were produced possessing an identical 5′ BNYVV-RNA3 sequence starting at position 750 and progressively shortened 3′ ends downstream of the “coremin” motif (Figure 4A). RNAs were treated with RppH (to generate a 5′ monophosphate) incubated with Xrn1 for 6 h, and residual RNA substrates and ncRNA products caused by Xrn1 stalling were detected by northern blot using a radiolabeled oligonucleotide complementary to the “coremin” sequence (Figure 4B). Residual unprocessed input substrate and ncRNA species of expected sizes generated by Xrn1 stalling were detected for 1R to 8R RNAs. Interestingly, the 9R RNA generated an ncRNA product that migrated slightly faster than expected (Figure 4B, lane 9R-RppH-Xrn1). We believe this was likely due to a particular folding of the ncRNA species that altered its migration in the acrylamide gel. Overall, these results indicated that all of the RNA structural elements necessary to stall Xrn1 are present within these 74 nt.

**Figure 5:**
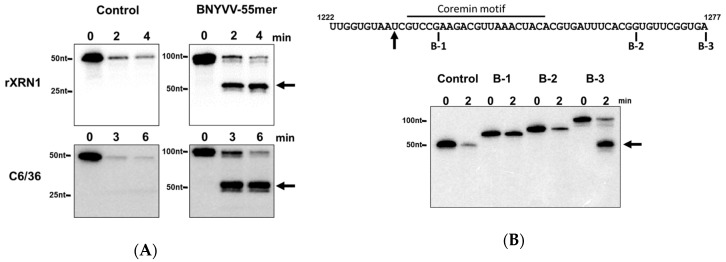
A 55 nt fragment containing the coremin motif is sufficient to stall XRN1. (**A**) 5′ monophosphorylated radiolabeled RNA substrates containing either control sequences derived from pGEM-4 or a 101-nt RNA containing a 55 base fragment from the RNA3 segment of BNYVV (nts 1222–1277) at the 3′ end and 53 nts of pGEM-4 polylinker sequence at its 5′ end to serve as a landing site for 5′-to-3′ exonucleases) were incubated with either purified recombinant XRN1 from *Kluyveromyces lactis* (rXrn1 panel) or cytoplasmic extract from C6/36 *Aedes albopictus* cells for the times indicated. Reaction products were resolved on a 5% acrylamide gel containing urea and viewed by phosphorimaging. (**B**) Top: sequence of the 55 nts BNYVV RNA fragment. The black arrow indicates the site of XRN1 stalling. Fragments B-1, B-2, and B-3 containing BNYVV-specific sequences ranging from position 1222 to the base indicated in the figure. Bottom: 5′ monophosphorylated radiolabeled RNA substrates containing either control sequences derived from pGEM-4 or the B-1, B-2, or B-3 fragments of the RNA3 segment (nts 1222–1277) as indicated in the top part of the panel (inserted into the pGEM-4 polylinker as indicated in panel A) were incubated with purified recombinant XRN1 for the times indicated. Reaction products were resolved on a 5% acrylamide gel containing urea and viewed by phosphorimaging. Arrows indicate the positions of the RNA species stalling XRN1.

We next complemented our mapping approach using in vitro transcripts with similar 5′ extremities starting at position 1222 of BNYVV RNA3 and possessing 16 (B-1), 44 (B-2), or 55 (B-3) nucleotides downstream in an Xrn1 decay assay performed using a short time course with an amount of highly active recombinant *Kluyveromyces lactis* Xrn1 enzyme similar to the amount of RNA substrate in the reaction as previously described [12,53]. As seen in Figure 5A,B, while a similar sized control RNA derived from pGEM-4 vector sequences failed to stall Xrn1 (at the site indicated by the arrow in Figure 5B), the 55 nt long ‘B-3’ transcript was able to effectively stall Xrn1. Shortening the BNYVV-derived sequences from the 3′ end by 10 (B-2) or more (B-3) bases completely abrogated the ability of the transcript to stall Xrn1 and generate a decay intermediate (Figure 5B). The 55-nt long BNYVV coremin-containing RNA was able to stall both recombinant Xrn1 (rXrn1) and Xrn1 derived from C6/36 insect cell extracts (Figure 5A), indicating that the domain is sufficient to stall Xrn1 from multiple sources. Thus, these data indicated that this 55 nt sequence (1222–1277) contained all of the RNA structural elements necessary to stall Xrn1. Combining the mapping data from Figure 4 and Figure 5 suggests that the minimal domain able to stabilize BNYVV ncRNA3 is likely 43 nt (1234 to 1277 nt). This domain includes two nucleotides preceding the “coremin” motif [33].

### 3.6. Accumulation of BNYVV ncRNA Represses Xrn Activity In Vitro

**Figure 6:**
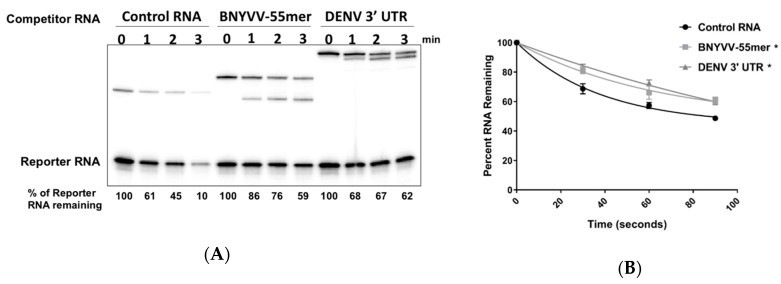
A 55 nt core fragment of RNA3 of BNYVV represses Xrn1. (**A**) A radiolabeled RNA containing a 5′ monophosphate (Reporter) was incubated with purified recombinant XRN1 for the times indicated. A 20X molar excess of lightly radiolabeled, 5′ monophosphorylated non-specific competitor RNA (‘Control RNA’ lanes), a competitor transcript containing the 55 nt core BNYVV RNA3 fragment (‘BNYVV-55mer’ lanes), or a competitor transcript containing the 3′ UTR of Dengue virus type 2 (‘DENV 3′ UTR’ lanes) was added to reactions. After the times indicated, reaction products were analyzed on 5% polyacrylamide gels containing urea and visualized by phosphorimaging. (**B**) Graphical presentation of the effect of the various competitor RNAs on Xrn1 activity on the Reporter transcript. Results shown are from three independent experiments. The asterisk represents a *p* value of <0.001 at both time points for viral 3′ UTR/BNYVV-55mer compared to the control as determined using a Turkey’s multiple comparisons test as a post-hoc test.

Previous work indicated that in addition to stalling Xrn1, structures from other viruses can also serve as reversible inhibitors and transiently but significantly repress Xrn1 activity [12,37,53]. Therefore, we next investigated the ability of the 55nt BNYVV domain to repress Xrn1 activity in vitro using the well-characterized Dengue virus (DENV) 3′ UTR element as a control. As seen in Figure 6A, while the presence of a 20X excess of a non-specific control RNA in the reaction had no effect on Xrn1 activity on a radiolabeled reporter RNA, the accumulation of ncRNA species due to Xrn1 stalling on either the BNYVV 55 nt RNA or the DENV 3′ UTR transcript correlated with an inhibition of the substrate degradation. Quantification of the rate of Xrn1 activity indicated that both the DENV 3′ UTR and the BNYVV 55nt domain repressed Xrn1 activity with similar kinetics (Figure 6B). Thus, we conclude that stalling of Xrn1 on BNYVV RNAs is also associated with repression of enzyme activity.

### 3.7. Exoribonuclease Stalling is Not Sufficient to Induce the Systemic Spread of the BNYVV in Beta macrocarpa

While ncRNA3 and ncRNA3sf are produced in a similar way in vivo and in vitro, we pursue a translational approach and test whether the production of ncRNA3sf could support the long-distance movement of BNYVV in *B. macrocarpa*. We inoculated *B. macrocarpa* leaves with RNA1 and 2 helper strain to ensure replication, encapsidation, cell-to-cell movement, and suppression of post-transcriptional gene silencing functions [30,33] alone or supplemented either with in vitro transcripts corresponding to RNA3 or RNA3E as described [30] or to chimeras RNA3sf or RNA3pk1. Inoculated leaves displayed local symptoms 7 days post-inoculation (dpi) and after 21 days, systemic infection was only observed in the presence of wt RNA3. No systemic infection was observed by symptoms, nor was virus detected by northern blotting, for plants inoculated with the helper strain supplemented with RNA3E, RNA3sf, or RNA3pk1 transcripts (Table 1).

**Figure 7:**
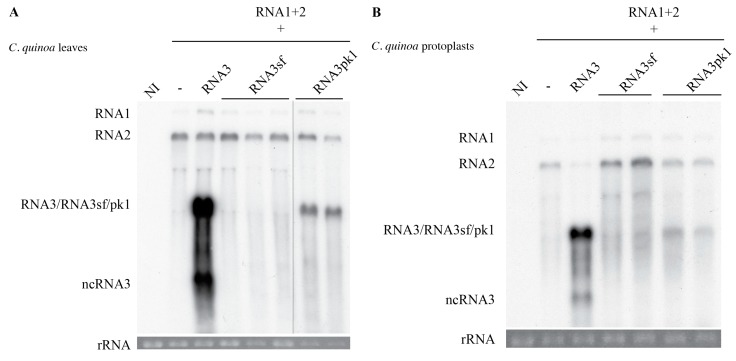
The BNYVV replication machinery does not replicate RNA3sf and poorly replicates RNA3pk1. *Chenopodium quinoa* leaves were rub-inoculated (**A**) or protoplasts electroporated (**B**) with RNA1+2 alone (-) or supplemented with RNA3, RNA3sf, or RNA3pk1. (**A**) RNA contents of local lesions were analyzed 7 days post-inoculation (dpi) by northern blot using BNYVV-RNA-specific radiolabeled probes. RNA3pk1 loading was three times lower than the other samples; the membrane was exposed accordingly and visualized with a split line. (**B**) Protoplasts contents were analyzed 40 h post-inoculation (hpi) as for (**A**). Loadings are visualized by ethidium-bromide staining of total RNAs (rRNA). NI, non-infected.

Northern blot analyses performed on infected *B. macrocarpa* inoculated leaves were similar to those presented for *C. quinoa* leaves or protoplasts (Figure 7) and revealed an absence of RNA3sf accumulation. Therefore, we were able to attribute such accumulation deficiency to the lack of the chimera’s replication, probably due to the highly structured sfRNA domain. However, RNA3pk1 species was able to accumulate in the infected leaves ~10 times lower than wt RNA3 without production of ncRNA3pk1 RNAs in the infected tissues or in protoplasts (Figure 7), suggesting its degradation in planta by an unknown process. Therefore, this approach did not allow us to draw conclusions about the direct implication of exoribonucleases in the efficient systemic movement of BNYVV.

### 3.8. Virus-Induced Gene Silencing of XRN4 Exoribonuclease Affects Viral Accumulation and Does Not Allow Systemic Spread in N. benthamiana

**Figure 8:**
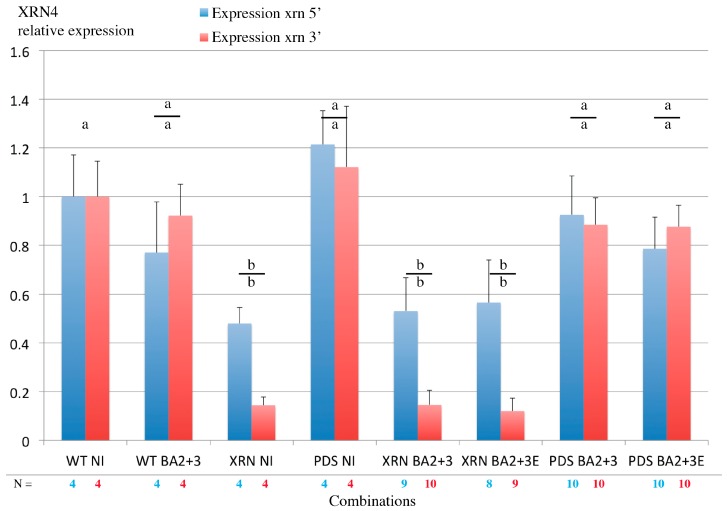
TRV-mediated VIGS of XRN4 is efficient in leaves. RT qPCR analyses of XRN4 mRNA within leaves from non-infected plants (WT NI), TRV-XRN (XRN), and TRV-PDS (PDS) silenced *N. benthamiana* leaves (NI) or rub-inoculated with RNA1+2BA2 supplemented with RNA3 (BA2+3) or RNA3E (BA2+3E). Non-silenced *N. benthamiana* inoculated with RNA1+2BA2 supplemented with RNA3 served as control (WT BA2+3). Values were normalized to a mock treated control plant (WT NI). Quantitative PCR was performed using specific primers targeting the 5′ (blue) and the 3′ (red) region flanking the XRN4 trigger sequence present in TRV RNA2 and GAPDH and PP2A cellular RNAs. The numbers of sampled plant are specified (N). Student test *p-*values were >0.005 (a) or <0.005 (b) using WT NI as reference (upper letter) or comparing 5′ and 3′ relative expression (lower letter).

Both tobacco rattle virus (TRV) and BNYVV infect *N. benthamiana*. TRV-mediated VIGS provides an efficient gene expression knockdown (KD). The effect of this KD on replication of BNYVV after inoculation was investigated bearing in mind a mixed infection context. While exoribonuclease XRN4 appears to be required for the production and accumulation of ncRNA3 species and ncRNA3 acts in synergy with BNYVV VSR [31], we tested the effect of a XRN4 KD on BNYVV long-distance movement. Such an approach was not applicable to wt BNYVV as the VSR is able to restore the expression of silenced genes [30]. Therefore, we used the BA2 hypomorphe mutant able to move a long distance only in the presence of ncRNA3 accumulation [31]. *N. benthamiana* plants were silenced using recombinant TRV constructs [54] carrying either the XRN4 sequence or the phytoene desaturase (PDS) sequence for the visualization of leaf photobleaching by PDS KD. Silenced leaves were inoculated with BNYVV-RNA1+RNA2BA2 [31] supplemented with RNA3 or RNA3E. Total RNAs were extracted both from inoculated and non-inoculated leaves. RTqPCR measured XRN4 expression while viral RNAs were detected by northern blot. Visual PDS KD was efficient in TRV-PDS infected plants as well as XRN KD. In BNYVV-inoculated leaves, depending on the region of mRNA considered, XRN4 mRNA levels were reduced to ~50% (probe 5′) up to ~90% (probe 3′) in XRN4 KD (Figure 8, compare XRN NI, XRN BA2+3 and XRN BA2+3E) while its expression did not significantly vary in PDS-KD-inoculated leaves (Figure 8, compare PDS NI, PDS BA2+3 and PDS BA2+3E). Both for PDS and XRN silenced plants, no BNYVV-BA2 mutant systemic movement was observed even in the presence of RNA3, probably due to interference between TRV and the BNYVV-BA2 mutant.

**Figure 9:**
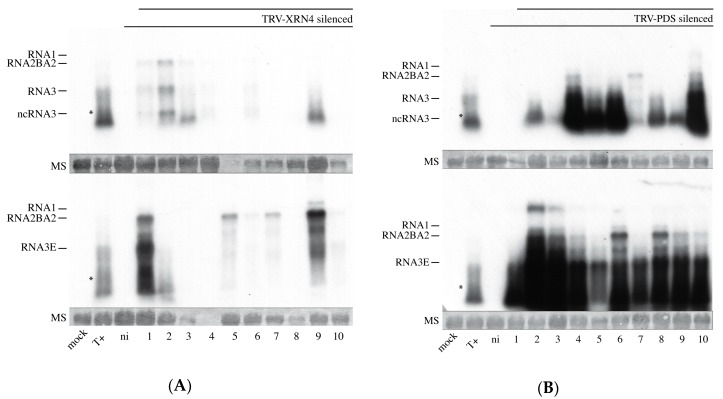
BNYVV RNA accumulation is compromised in XRN4 KD plants. TRV-XRN- (**A**) and TRV-PDS- (**B**) silenced *N. benthamiana* plants were rub-inoculated with RNA1+2BA2 supplemented with RNA3 (top) or RNA3E (bottom). Equivalent amount of total RNAs (~10 µg, visualized by methylene blue staining, MS) were extracted from the inoculated leaf of each plant (1–10) and analysed by northern blot using specific probes for BNYVV RNA1, 2, and 3. Mock, control plant; T+, *N. benthamiana* infected with RNA1+2BA2+RNA3; ni, TRV-silenced and non-inoculated with BNYVV. The poor quality of the northern blots is due to mixed TRV and BNYVV infection. Samples in panel A lanes 5 (top) and 4 (bottom) were lost. The asterisk indicates the position of ncRNA3. Upper bands of panel B (e.g., lanes 2 and 3) are of unknown origin and may correspond to uncompleted denaturation of viral RNAs. Panel A and B membrane treatments were simultaneous using the same combination and amount of probes. Both blots were exposed 120 h to allow the visualization of viral RNAs of panel A.

Viral RNAs were detected in 6 inoculated leaves out of 10 in XRN4 KD plants (Figure 9A) and 9 and 10 inoculated leaves out of 10 in PDS KD plants (Figure 9B). Interestingly, the accumulation levels of viral RNAs in the leaves were strikingly different. In XRN4 KD leaves, BNYVV RNAs were poorly accumulated compared to PDS KD control plants (compare Figure 9A,B, exposition for XRN KD is 2.5 times higher than PDS KD). Despite an efficient KD of XRN4, ncRNA3 was accumulated (Figure 9A,B, top panels).

## 4. Discussion

Viral long-distance movement within the *B. macrocarpa* host requires the cis-active RNA sequence named ‘core’ and is not based on RNA3 p25 expression [34]; it involves a synergistic action with the p14 VSR [31]. Within ‘core’ stands the “coremin” motif [35], a 20 nt sequence also found in the 3′ UTR of other viral species [33]. Such a “coremin” motif is involved in the stabilization of ncRNA3 after nuclease processing and the systemic infection of *B. macrocarpa* species. Indeed, mutations introduced within “coremin” prevented ncRNA3 species accumulation and viral systemic movement on *Beta* species [33]. The use of host factors KO mutants is the most elegant approach to elucidate pathways involved in biological processes. If KO mutants are easily obtained in *A. thaliana*, such a plant is unfortunately not a host for BNYVV and therefore not suitable to set up genetic screens. Furthermore, *B. macrocarpa* is far from being genetically characterized. Therefore, other approaches consist in the use of heterologous genetic systems able to recapitulate one or more biological functions. We expressed BNYVV-RNA3 in *S. cerevisiae* outside a context of viral infection. We succeeded in the detection of ncRNA3, indicating the presence of yeast cellular factor recapitulating plant processes. Using *S. cerevisiae* KO mutants, we identified Xrn1 exoribonuclease as the main actor for ncRNA3 production from ectopically expressed RNA3 species, which was confirmed by complementation experiments of the *S. cerevisiae ∆xrn1* mutant strain with plant XRN4 or yeast Xrn1 (Figure 1).

Xrn1 stalling leads to flavivirus sfRNA production thanks to a ternary three-way junction structure [8,13]. We replaced the BNYVV-RNA3-core sequence with the WNV domain required for the flavivirus ncRNA accumulation and reproduced the data published for this animal virus (Figure 2), with a slight difference within the 5′ extremity of sfRNA that appeared to be extended by 3 nt in our hands as compared with previous primer extension analyses [9]. Beyond reproducing previous data, we further showed that plant XRN4 is well-inhibited by flavivirus structures in yeast. We used in vitro degradation studies with purified Xrn1 and chimeric RNA3sf/BN3sf RNA substrates and reproduced Xrn1 stalling (Figure 3). We applied a similar method [12] to demonstrate that ncRNA3, through its 20 nt “coremin” sequence, stalls in vitro RNA processing by Xrn1, leading to ncRNA3 accumulation. These data did not demonstrate a clear inhibitory effect of ncRNA3 on Xrn1, since ncRNA3 accumulation plateaued while full-length species continued to be processed (Figure 4). However, using a parallel approach, we proved that ncRNA3 blocks Xrn1 activity similar to flavivirus noncoding RNAs (Figure 6). These experiments also demonstrated that while ncRNA3 production appears to be independent of the 5′ sequence upstream of the “coremin” motif (Figure 3 and Figure 5), the minimal length required for exoribonuclease stalling involves the “coremin” motif followed by an RNA3 sequence of 21 nt (Figure 4 and Figure 5).

The RNA3sf species failed to accumulate efficiently in inoculated protoplasts or plant leaves for unknown reasons and could not be used for gain-of-function experiments to allow the systemic spread of BNYVV on *B. macrocarpa*. If the RNA3pk1 species could be replicated in the presence of the RNA1+2 helper strain, it accumulated about ten times less than wt and without production of ncRNApk1 (Figure 7). Interestingly, RNA3 mutant species harboring the “coremin” motif and replicating at low levels still provide systemic movement in *Beta* species (e.g., t35∆SSt in [34]). We suspect that the absence of ncRNA3pk1 species detected in yeast and in vitro (Figure 2 and Figure 3) could explain the lack of viral long-distance movement (Table 1). Therefore, these domains appear apparently not interchangeable in the viral context although both act on exoribonucleases.

Virus-induced gene silencing (VIGS) knockdown is a well-described method for functional validation of cellular pathways required in biological processes. This approach requires a systemic plant infection with a non-related virus possessing a sequence derived from the target gene. Reduction of XRN4 expression was not sufficient to alleviate the production of the ncRNA3 species. The ncRNA3 was still detected (Figure 9A top panel) with genomic RNA accumulation considerably reduced in XRN4-KD and not in PDS-KD leaves, suggesting either the requirement of XRN4 in the BNYVV replication cycle or pleiotropic effects due to the global disruption of mRNA turnover. Moreover, the presence of a TRV/BNYVV mixed infection was in favor of RNA1+2BA2+RNA3E accumulation (Figure 9, compare lane T+ with lanes 1–10). This behavior is in opposition with previous results, where RNA1+2BA2+RNA3 accumulated to higher levels than an inoculum-containing RNA3E mutant [31]. Here, we suspect an effect of the TRV infection on the BNYVV accumulation that appears not to be compromised by ncRNA3 accumulation. Such behavior could as well explain the absence of the long-distance movement of BNYVV-BA2 variant RNA species.

## 5. Conclusions

Our experiments demonstrate the role of the “coremin” sequence and 3′ proximal sequences in 5′-to-3′ exoribonuclease stalling leading to the accumulation of ncRNA species and to long-distance movement of the virus. In the context of VIGS mixed infection, no viral long-distance movement was observed even when a PDS control was used. Such absence of systemic movement is not explained and could be the result of the coinfection of *N. benthamiana* with TRV and BNYVV. XRN4 KD lowered BNYVV RNAs accumulation, suggesting a role of the nuclease in the replication steps of BNYVV.

Our results can be correlated to those obtained with bamboo mosaic virus, whose replication complex hijacks the XRN4 ribonuclease to ensure an efficient amplification of the virus [26]. West Nile virus, tobacco mosaic virus, and red clover mosaic virus interfere as well with XRN-mediated pathways to facilitate viral replication and to favor the infection [9,16,19], and the exact role of the nuclease in the biology of these viruses needs to be characterized. While ncRNAs production regulates the viral cycle of these viruses, the BNYVV long-distance movement complementation is provided by RNA species containing the “coremin” motif, such as BNYVV-RNA5 or BSBMV-RNA3. Therefore, we suspect a direct role for this “coremin” sequence that could possibly act as a recognition motif as a key component of the viral long-distance movement complex.

## Figures and Tables

**Table 1:** Effect of the RNA3-derived species on the long-distance movement of BNYVV in *Beta macrocarpa.*

Combinations	1st Experiment	2nd Experiment
RNA1+2	0% (5)	0% (5)
RNA1+2+3	70% (10)	90% (10)
RNA1+2+3E	0% (10)	NT
RNA1+2+3sf ^†^	0% (10)	0% (10)
RNA1+2+3pk1	0% (10)	0% (10)

Plants were inoculated with BNYVV-RNA1+2 alone or supplemented with RNA3, RNA3E, RNA3sf, or RNA3pk1. Viral RNAs detection on systemic leaves was performed 21 days post-inoculation. Percentage of plants infected systemically (total infected plants); NT, not tested. †: In previous experiments, plants showed systemic symptoms that were due to an RNA3 contamination of unknown origin.

## Supplementary Materials

The following are available online at http://www.mdpi.com/1999-4915/10/3/137/s1, Figure S1: Expression of BNYVV RNA3 species in *Saccharomyces cerevisiae* FY4 and mutated strains.
