# Supplementary material for: Beet Necrotic Yellow Vein Virus Noncoding RNA Production Depends on a 5′→3′ Xrn Exoribonuclease Activity
Source: Viruses. 2018 Mar 19;10(3):137. doi: 10.3390/v10030137 (PMC5869530; doi:10.3390/v10030137)
Supplement: Supplementary file 1 [file viruses-10-00137-s001.pdf]

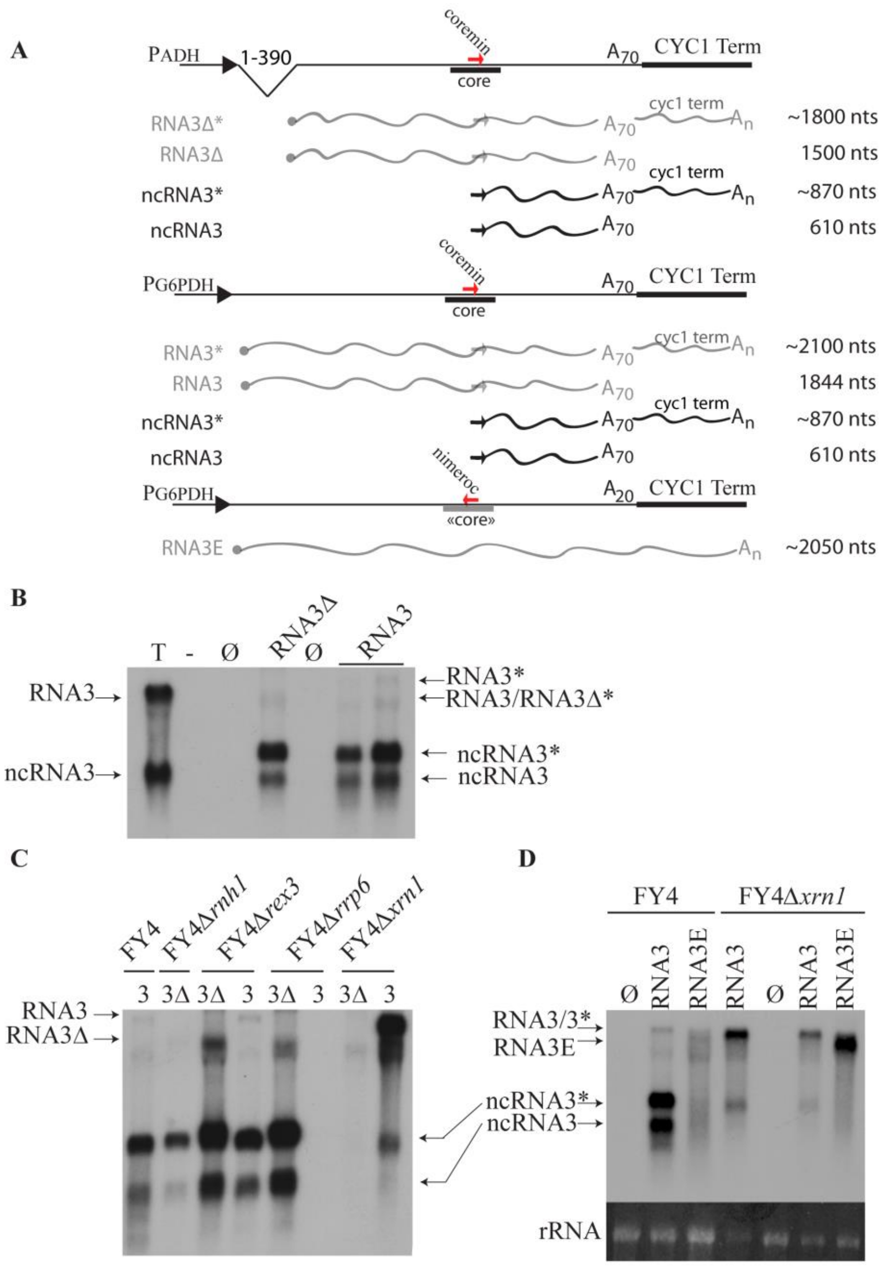

Figure S1. Expression of BNYVV RNA3 species in *Saccharomyces cerevisiae*. (**A**) Drawing of the expression vectors used to transform yeasts. ADH and G6PDH promoters depicted as black arrows drive the transcription of RNA3 or RNA3 truncated (Δ) of its 5′ UTR that appears as a broken line. The "coremin" motif and its reverse complement sequence are represented as sense and antisense red arrows. Grey filled circle corresponds to the cap and A_{70} and A_{20} correspond to the number of Adenosine residues. CYC1 term is the terminator sequence. A_n corresponds to the polyA tail added *in vivo.* Sizes of the RNA species are depicted. RNA3* differs from RNA3 by the presence of the cyc1 terminator and polyA sequences -See Fig1C - (**B-D**). RNA3 species were detected by northern blotting of total RNAs issued from wild-type FY4 yeast (B and D) and nucleases mutants FY4Δ*rhn1*, FY4Δ*rex3*, FY4Δ*rrp6* (C) and FY4Δ*xrn1* (C and D). The probe was complementary to the 3′ UTR of RNA3. (-) unloaded; Ø, empty vector 5 µg of RNA were loaded (panel C to D). Ethidium bromide staining is only available for panel D (rRNA). T corresponds to RNAs issued from BNYVV infected plant and is used as a position marker (B).
